# Supplementary material for: Mechanism and resistance for antimycobacterial activity of a fluoroquinophenoxazine compound
Source: PLoS One. 2019 Feb 22;14(2):e0207733. doi: 10.1371/journal.pone.0207733 (PMC6386362; doi:10.1371/journal.pone.0207733)
Supplement: S1 Fig — (PDF) [file pone.0207733.s001.pdf]

A

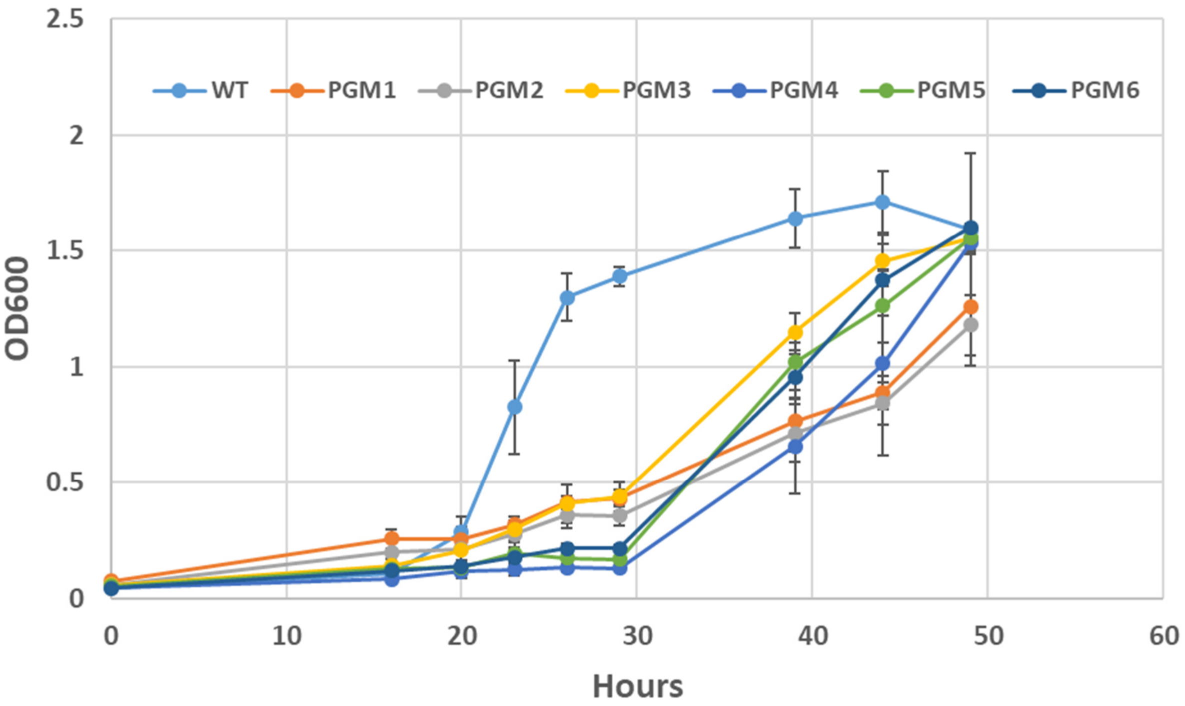

B

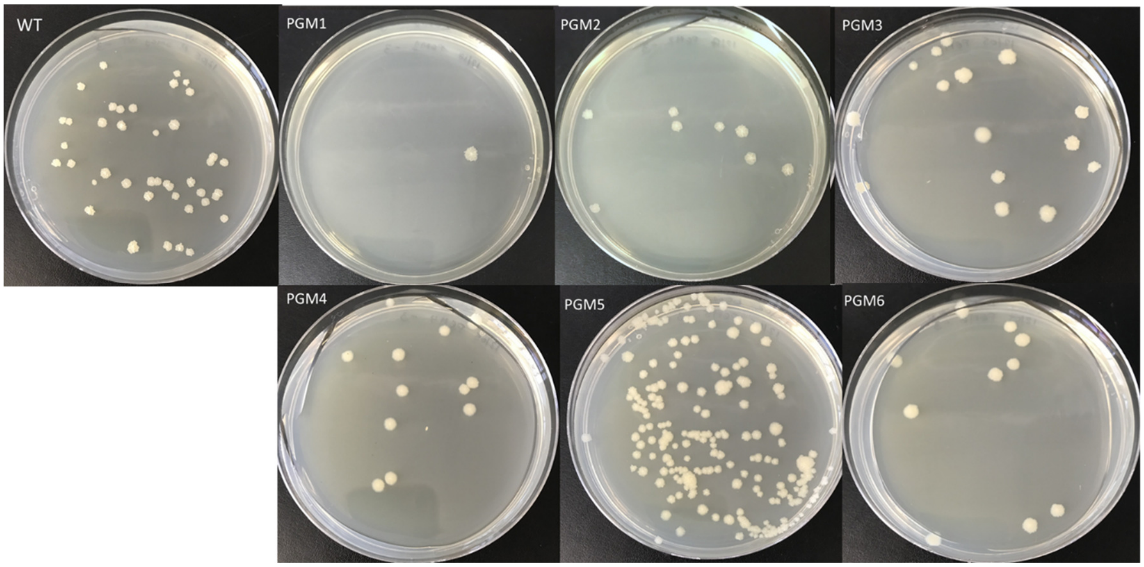

S1 Fig. Effect of FP-11g resistant mutations on *M. smegmatis* growth and colony morphology. (A) WT (mc2 155) and FP-11g resistant mutants (PGM1-PGM6) overnight cultures were adjusted to an optical density (OD600) of 0.1 and subsequently diluted 1:10 using 7H9 growth media with ADN supplement. Fifty microliters (~10^5 cfu) of the diluted culture were transferred to the individual wells of a clear round-bottom 96-well plate containing 50 µl of serially diluted compounds. The 96-well plate was then incubated at 37°C with shaking. OD600 readings were recorded at the indicated time points with the Biotek Synergy II plate reader. The growth curves represent the average and standard deviation from three experiments. (B) Colony morphology of WT (mc2 155) following 4 days of incubation or FP-11g resistant mutants (PGM1-PGM6) following 7 days of incubation from diluted cultures spread on LB plates.
