# Supplementary material for: Mechanism and resistance for antimycobacterial activity of a fluoroquinophenoxazine compound
Source: PLoS One. 2019 Feb 22;14(2):e0207733. doi: 10.1371/journal.pone.0207733 (PMC6386362; doi:10.1371/journal.pone.0207733)
Supplement: S1 Table — (PDF) [file pone.0207733.s002.pdf]

S1 Table. Summary of mutations associated with FP-11g resistance in *M. smegmatis*

Gene	Homolog in *Mtb*	Region	Freq	Type	Nucleotide		Amino acid		Functional annotation in *M. smegmatis*	Functional annotation in *M. tuberculosis*
					Ref	Allele	Ref	Allele		
**MSMEG_0965**	No	1039098	**4**	SNV	C	T	T	I	Porin MspA	N/A
**MSMEG_0965**	No	1039173	**2**	SNV	T	C	L	P	Porin MspA	N/A
**MSMEG_2820**	No	2883459	**1**	SNV	T	G	I	S	Hypothetical protein. Function unknown. Integral component of membrane	N/A
**MSMEG_6430**	No	6498888^6498889	**3**	INS	-	C	S	fs	Hypothetical protein. Function unknown. Integral component of membrane	N/A
**MSMEG_6430**	No	6498891^6498892	**2**	INS	-	C	D	fs	Hypothetical protein. Function unknown. Integral component of membrane	N/A
**MSMEG_6430**	No	6498895	**2**	SNV	G	C	S	C	Hypothetical protein. Function unknown. Integral component of membrane	N/A
**MSMEG_6430**	No	6498898^6498899	1	INS	-	T	P	fs	Hypothetical protein. Function unknown. Integral component of membrane	N/A
**MSMEG_6430**	No	6498901	2	SNV	G	T	T	K	Hypothetical protein. Function unknown. Integral component of membrane	N/A
**MSMEG_0241**	Rv0202c	270553^270554	**2**	INS	-	C	L	fs	MmpL11 protein. Function unknown	unknown/cell wall and cellular processes
**MSMEG_5623**	No	5707525	**3**	SNV	C	A	D	Y	L-carnitine dehydratase. Function unknown	N/A
**MSMEG_0933**	Rv0486	1013796	**1**	DEL	C	-	R	fs	Conserved hypothetical protein. Function unknown	*mshA* gene: Glycosyltransferase
**MSMEG_0240**	Rv0201c	269640^269641	**1**	INS	-	A	M	fs	Conserved hypothetical protein. Function unknown	conserved hypothetical /unknown
**MSMEG_1513**	No	1604708	**2**	SNV	G	C	S	C	Conserved hypothetical protein. Function unknown. oxidoreductase activity	N/A
**MSMEG_4629**		4715711^4715712	**1**	INS	-	T			pseudogene	N/A
**MSMEG_3552**	No	3612348	**1**	SNV	C	G	E	Q	Conserved hypothetical protein. Function unknown	N/A

Freq: frequency; SNV: single nucleotide variation; INS: insertion; DEL: Deletion; Ref: reference; fs: frame shift
